# Organization-driven research: how anticipated reputational risk shapes organizational influence on knowledge production in sensitive organizational research

**DOI:** 10.3389/fpsyg.2026.1869979

**Published:** 2026-07-31

**Authors:** Katharina Kärgel

**Affiliations:** School of Health, Education and Social Sciences, SRH University, Heidelberg, Germany

**Keywords:** field access control, gatekeeping, knowledge production, obedience, organization studies, organizational impression management, organization-driven research, sensitive topics

## Abstract

While organizational research on sensitive issues often acknowledges gatekeeper control and power asymmetries, these dynamics are typically framed as isolated external constraints or logistical hurdles affecting access and data quality. This perspective does not adequately capture the role of organizations in systematically shaping the research process in its entirety. Following a phenomenon-based theorizing approach, this paper aims to address this gap by introducing the concept of *organization-driven research*, defined as a distinct phenomenon through which organizations actively shape and delimit research on sensitive issues. The paper draws on 64 semi-structured interviews, fieldnotes, and documented gatekeeper communications collected as part of a qualitative study on destructive workplace obedience conducted within police, military, and university hospital settings in German-speaking countries. The findings reveal three interdependent mechanisms of organization-driven research. First, *control over access and information* is exercised through strategic gatekeeping and hierarchical participant pre-selection, which embeds participants in relations of accountability. Second, *control over the research topic’s perspectivation* involves the depoliticization of sensitive topics and narrative control through which certain interpretations are privileged while others are marginalized. Third, *control over research design* manifests through the modification or restriction of methods previously agreed upon, limiting researcher autonomy over data collection. Accordingly, the paper theorizes organization-driven research as a manifestation of *organizational impression management* through which organizations seek to regulate how sensitive organizational realities are represented to external audiences, mitigate anticipated reputational risks, and maintain organizational legitimacy. The paper offers a reflexive framework that treats relational dynamics and narrative inconsistencies as analytically meaningful indicators of organizational power relations, and provides methodological guidance for conducting research in settings where organizational influence is likely to shape access, participation, and disclosure. In doing so, the study reconceptualizes organizational influence from a methodological challenge to a constitutive condition of knowledge production in organizational research.

## 1 Introduction

Organizational research is marked by a fundamental divergence of interests: organizations and their members may pursue goals that are opposed to those of researchers (Cunliffe and Alcadipani, 2016), while the interests of individual members may simultaneously conflict with those of the organization itself (Coupal, 2005). Cunliffe and Alcadipani (2016, p. 536) therefore conceptualize organizational access as a “political process of immersion, backstage dramas, and deception,” characterized by uncertainties, complexities, and evolving circumstances that require ongoing (re)negotiation and strategic consideration by researchers (Americo et al., 2024; Aroles, 2020; Bondy, 2013; Saharan et al., 2021). Gatekeepers’ decisions to approve or deny research projects are accordingly tied to organizational priorities, which in turn determine the extent and intensity of organizational control and resistance exercised throughout the research process (Crowhurst and Kennedy-Macfoy, 2013; McCarthy and Lee, 1995; Stefan et al., 2024; Stevens, 2020).

Research on issues that are perceived as sensitive, controversial, or potentially threatening to the organization tends to encounter more pronounced forms of control and resistance (Adu-Ampong and Adams, 2020; Dempsey et al., 2016; Dickson-Swift et al., 2006). In organizational settings, such sensitivity often arises when an organization’s association with certain behaviors or incidents is perceived as “socially undesirable (and sometimes legally prosecutable) for employees or for organizations as entities” (King et al., 2013, p. 504).

Accordingly, gatekeepers perform a dual function: they facilitate research by granting legitimacy, identifying relevant participants, and navigating internal structures (Crowhurst and Kennedy-Macfoy, 2013), while simultaneously acting as organizational representatives tasked with safeguarding organizational integrity, autonomy, and reputation (Kay, 2019). From the perspective of institutional gatekeepers, researchers represent “a relatively uncontrolled element within an otherwise highly controlled environment,” requiring careful assessment and negotiation of potential risks or adverse outcomes (Gřundělová et al., 2025, p. 637). This is reflected in a variety of formal and informal practices through which gatekeepers simultaneously enable and regulate access to the field, ranging from overt interventions to more subtle forms of influence; they filter information, oversee interactions between researchers and organizational members, and selectively disclose data that is considered safe for the organization’s image (Broskevičová et al., 2024; Kay, 2019; Savolainen, 2020). More indirect resistance tactics include the tendency of gatekeepers to deflect responsibility, regulate communication channels, repeatedly request additional information, or fail to honor prior agreements (Fecke et al., 2022; Stonebanks et al., 2019; Wanat, 2008). Thus, gatekeepers can define the problem area under investigation, limit access to data and participants, restrict the scope of analysis, or retain influence over the publication of findings (Broadhead and Rist, 1976; Clark, 2011; Fitz-Gibbon, 2016).

Yet, structural factors such as limited organizational resources, low prioritization of research activities, or limited familiarity with research processes are often cited as justification for restricted access and organizational engagement (Brodeur et al., 2025). However, such rationales frequently serve as a polite front for a deeper discomfort and an unwillingness to expose the organization to public scrutiny—particularly where critical research could reveal internal conflicts, challenge institutional legitimacy, or entail reputational risks by exposing the organization to uncontrolled participant disclosure (Fecke et al., 2022; Gřundělová et al., 2025; Stonebanks et al., 2019).

Research on sensitive issues not only intensifies gatekeeper control, but can also evoke feelings of shame, guilt, and fear of exposure or retribution among participants (Lee and Renzetti, 1990), thereby giving rise to well-documented ethical and methodological challenges throughout the research process (Silverio et al., 2022). It is well established that socially undesirable behaviors and attitudes tend to be underreported (Krumpal, 2013; Momenpour Taula et al., 2022). For instance, participants may position sensitive issues “so far outside the boundaries of reality as to be not worth considering” (Enosh and Buchbinder, 2005, p. 594) or strategically adjust their accounts to correct perceived misrepresentations or polish up their personal or social image (Jacobsson and Åkerström, 2013).

These dynamics are crucial in workplace settings where disclosures involve personal, professional, and institutional risks. Employees often weigh rewards against risks to their professional identity, employment security, and social standing when deciding whether to reveal sensitive information (Jehn and Jonsen, 2010; Linstead et al., 2014; Morrison and Milliken, 2000). While such careful weighing is often linked to limited trust in the research context (Baum-Talmor, 2020; Othman and Abdul Hamid, 2018), Scheytt and Pflüger (2024) argue that it is fundamentally rooted in organizationality—the structural characteristics of organizations (i.e., formal hierarchy, formal membership, sanction mechanisms, etc.). In hierarchical contexts, voluntariness may be compromised, making informed consent more formal than substantive (Resnik, 2024). The expectation of organizational returns may challenge the ethical guarantees of anonymity and confidentiality, particularly when organizational insiders can infer the identities of participants based on contextual knowledge (Armenakis et al., 1975; Czarnota-Bojarska, 2021). Participants in subordinate positions thus face what Scheytt and Pflüger (2024) call structural harm: they are vulnerable to risks associated with disclosure, including sanctions within organizational hierarchies. As a result, researchers may encounter data that are filtered, contradictory, exaggerated, or selectively disclosed, depending on levels of trust and the conditions under which data collection takes place (KC and Dahal, 2025; Othman and Abdul Hamid, 2018).

To navigate the methodological and ethical challenges inherent in conducting research on sensitive issues within organizational settings (Scaratti et al., 2021), researchers frequently find themselves compelled to adopt adaptive strategies. These strategies may include modifying research questions, adjusting designs, reducing sample size, or accommodating organizational expectations (Cunliffe and Alcadipani, 2016; Americo et al., 2024). While such adaptations are typically framed as necessary pragmatic adjustments to enable data collection (Buchanan and Bryman, 2007), they are also epistemically consequential. Existing literature tends to address the outlined challenges as isolated, manageable issues that can be mitigated through credibility management, relationship-building, anticipation of organizational concerns, or negotiation with organizational actors (Americo et al., 2024; Crowhurst and Kennedy-Macfoy, 2013; Kay, 2019; Monahan and Fisher, 2015).

Although the aforementioned strands of literature document crucial aspects of organizational influence on research trajectories, they remain analytically fragmented, addressing such dynamics as isolated methodological challenges rather than as manifestations of systematic organizational influence on the research process. As Gřundělová et al. (2025, p. 624) observe, there are still “only few published studies in which researchers have discussed the control that gatekeepers exerted when forcing researchers to either revise or depoliticize their analysis.” In particular, it remains theoretically underexplained how individual challenges such as gatekeeping, access restrictions, methodological interference, or participant self-censorship interact to constitute a broader pattern of organizational influence. Equally underexplained is what accounts for the apparent discrepancy between organizations’ formal approval of research and their subsequent systematic efforts to shape or constrain it.

Following the approach of phenomenon-based theorizing (Von Krogh et al., 2012), this paper aims to address this gap by examining the following questions: What are the defining characteristics of systematic organizational influence on the research process in sensitive organizational research, and how can this phenomenon be conceptually captured? What underlying organizational logic accounts for the discrepancy between formal research approval and subsequent organizational efforts to shape or constrain the research process? What methodological implications follow from understanding organizational influence as a constitutive condition of knowledge production?

To address these questions, the paper develops the concept of *organization-driven research* to capture how organizations act as active agents in shaping and delimiting research processes. Drawing on field experiences from a study of destructive workplace obedience, organization-driven research is conceptualized as a distinct phenomenon that links previously fragmented observations of organizational influence into a coherent analytical framework. Drawing on *organizational impression management*, the paper theorizes organization-driven research as an organizational effort to regulate external representations of sensitive organizational realities and maintain institutional legitimacy.

These considerations yield three key contributions. *Theoretically*, the paper introduces organization-driven research as a distinct phenomenon that extends existing conceptualizations of gatekeeping by foregrounding systematic organizational involvement in shaping research processes. *Epistemologically,* the paper demonstrates how organization-driven research can be understood as a manifestation of organizational impression management, thereby showing how organizational involvement in research processes systematically shapes the production and interpretation of knowledge about organizations. *Methodologically*, the paper outlines how to anticipate and navigate organization-driven research in studies of sensitive organizational phenomena.

The paper is structured as follows. Section 2 presents the research context. Section 3 describes the analytical approach. Sections 4 and 5 present the empirical manifestations of ODR and their theoretical interpretation as organizational impression management. Section 6 discusses theoretical, epistemological, and methodological implications, limitations, and future research directions.

## 2 Research context

The concept of *organization-driven research* (ODR) emerged inductively during a research project originally designed to investigate destructive workplace obedience, understood as situations in which employees comply with authoritarian orders that raise moral, ethical, or legal concerns (Lepage et al., 2019; Milgram, 1963). Consistent with the serendipitous nature of qualitative inquiry, recurrent field experiences revealed forms of organizational influence that were not anticipated in the original research design. This led to a theoretical reorientation that aligns with the logic of phenomenon-based theorizing (Fine and Deegan, 1996). These dynamics became visible precisely because the study combined a sensitive research topic with organizational contexts characterized by hierarchy, accountability, and researcher dependence on organizational access.

The study was conducted in three organizational settings—police, military institutions, and university hospitals—in German-speaking countries. The selection of this regional context reflects pragmatic considerations of field access. At the same time, the selection of German-speaking countries reduced variation arising from broader cultural, linguistic, and institutional differences, thereby facilitating comparison across organizational settings. The organizational settings, however, were selected for theoretical and analytical reasons. First, they are characterized by formalized authority structures, hierarchical accountability, and bureaucratic control mechanisms (Bendor and Swistak, 2025), making patterns of organizational influence particularly pronounced and empirically traceable. Second, all three operate in fields where institutional image is not merely strategically desirable but constitutive of their public legitimacy and social mandate (Brady and Schlozman, 2022; Lock and Jacobs, 2025), rendering research encounters organizationally consequential. Third, the consideration of institutions typically associated with authority, discipline, and the potential use of force (police and military) alongside settings more commonly perceived as prosocial (hospitals) (Blaskovits et al., 2022; Etzioni, 1975; Hibbard et al., 2005) provides analytical value. The recurrence of similar dynamics across organizations with markedly different societal mandates suggests that ODR may reflect structural characteristics of organizational settings rather than the specific mission of any organization.

## 3 Analytical approach

As outlined above, organization-driven research emerged inductively from observations that were not anticipated in the original study design. To develop a conceptual understanding of these observations, the present study adopts a phenomenon-based theorizing approach. The following sections describe the empirical material, analytical procedures, theory development process, and reflexive considerations underlying the conceptualization of ODR.

### 3.1 Data and sample

Given that the original study investigated destructive workplace obedience rather than organizational influence on the research process itself, the manifestations of ODR emerged from research encounters not designed to elicit them. This constitutes both a boundary condition and an analytical strength, reducing the likelihood that participants responded to researcher expectations regarding ODR.

The original research design combined semi-structured interviews with a role-play. Although the role-play was formally approved, it could not be fully realized in practice and was repeatedly modified, postponed, or ultimately not implemented. The data for the present paper therefore include semi-structured interviews (*N* = 64), fieldnotes, postscripts, and systematic documentation of gatekeeper communications. Postscripts and fieldnotes, produced immediately following each field encounter, systematically documented interactional dynamics and researcher observations that fell outside the formal interview record.

Access was primarily granted through formal organizational gatekeepers. Since formal channels were organized hierarchically and thus limited independent contact with potential participants, additional recruitment was pursued through informal and snowball sampling methods (e.g., calls to action in newsletters from labor unions or professional associations).

The final sample comprised employees from police (*n* = 21), military (*n* = 21), and university hospitals (*n* = 22). Of these, 32 participants occupied leadership roles, 22 non-leadership roles, and 10 intermediate positions characterized by both supervisory and subordinate responsibilities. Intermediate positions were particularly common in the military setting, where participants frequently exercised authority over subordinates while simultaneously reporting to higher-ranking superiors. Of the 64 interviewees, 33 were recruited through formal requests and 31 were recruited through informal channels. All participants provided written informed consent; anonymity and confidentiality were ensured throughout, with all identifying personal data replaced by pseudonyms.

### 3.2 Analytical process and theory development

The analysis proceeded in two phases reflecting the serendipitous origin of ODR. First, data were analyzed using MAXQDA 2024 following a Grounded Theory approach (Glaser and Strauss, 1967) to address destructive workplace obedience. Memos documented emerging interpretations and theoretical connections.

The second phase emerged from the observation that recurring field experiences—such as patterns of participant pre-selection, resistance to research methods, and communicative strategies in interviews—could not be adequately captured within the original research questions. Through iterative comparison, recurring patterns were grouped into three domains of organizational influence: (1) control over access and information, (2) control over the perspectivation of the research topic, and (3) control over the research design. Competing interpretations were documented in analytical memos and revisited through iterative re-engagement with the raw data. This reanalysis followed an inductive-interpretive logic consistent with phenomenon-based theorizing, moving iteratively between empirical observations and theoretical interpretations (Fisher et al., 2021; Von Krogh et al., 2012; van de Ven, 2016).

As these categories were refined through iterative analysis, concerns regarding organizational image, legitimacy, and external representation emerged as a connecting logic. Organizational impression management was thus introduced to interpret the underlying logic of ODR.

### 3.3 Reflexivity and alternative explanations

The analytical process was shaped by the researcher’s own fieldwork experience: initial recognition of organizational influence patterns preceded their systematic analysis and was informed by situated encounters during data collection. Consistent with qualitative research traditions, this experiential grounding is treated as an epistemic resource that enabled attentiveness to dynamics that might otherwise have remained analytically invisible (Kühner et al., 2016).

Initially, the dynamics that would later constitute ODR were experienced as practical and analytical frustrations: agreements not honored, access constrained after being granted, and participants who seemed to present themselves and their profession in a favorable light rather than engaging openly with the research encounter. It was also initially unclear how to handle the contradictions between on- and off-record accounts, or whether such material was analytically usable at all. The gradual recognition that these experiences formed a coherent pattern—rather than incidental fieldwork complications—was enabled by the researcher’s outsider position: without prior membership in these organizational contexts, what might have gone unnoticed by an insider became analytically visible precisely through its contrast with the researcher’s expectations.

Thus, the analysis took seriously the risk of over-attributing organizational control intent. Alternative explanations—including bureaucratic contingencies, resource constraints, or individual participant behavior—were actively considered through negative case analysis (Lincoln and Guba, 1985) and compared against patterns across all three organizational contexts. Cross-contextual coherence served as the primary criterion for theoretical consolidation.

Organizational impression management is therefore advanced as one valuable interpretation of ODR rather than its exclusive explanation: it was retained because it accounted for the cross-contextual coherence of ODR dynamics, the timing of organizational interventions relative to methodological specificity, and the systematic correspondence between the type of control observed and the reputational risks involved. The credibility of the analysis is further supported by cross-case comparison across three organizationally distinct settings, which function as a form of internal triangulation. Because the study was conducted by a single researcher, the analysis relied on reflexive memoing, iterative re-engagement with raw data, and retention of inconsistencies and contradictions as analytically meaningful rather than smoothing them.

## 4 Manifestations of organization-driven research

In the engagement with the data material, organizational influence is evidenced through (1) control over access and information, (2) control over the research topic’s perspectivation, and (3) control over the research design. In line with phenomenon-based theorizing, these manifestations constitute *organization-driven research.* The manifestations of this phenomenon are presented as a proof-of-concept rather than an exhaustive classification. They are first discussed individually before being analyzed in their interrelations as a cascading process of organizational control.

### 4.1 Control over access and information

Control over access and information emerged as a central mechanism through which organizations shaped the research process. Rather than merely enabling or restricting entry, organizations structured who became available as participants, under which conditions interactions took place, and what prior information framed these encounters. What initially appeared as incidental fieldwork complications was, through recurring similarities across organizational settings, increasingly recognized as a coherent pattern of motivated control.

#### 4.1.1 Strategic gatekeeping and participant pre-selection

A central manifestation of control over access and information was gatekeeper-driven participant selection, which was already constrained at the level of ministerial or top organizational leadership. For instance, access to police departments and military units was pre-structured through prior selection processes, and requests to include additional districts or units were not granted despite repeated inquiries and without formal explanation being provided.

At the level of formal gatekeeping, gatekeepers—consistently superiors positioned one hierarchical level below those who granted official research approval—were themselves eligible as participants and also functioned as intermediaries for recruiting further interviewees. Rather than merely facilitating access, they actively curated the sample: participants were frequently selected based on organizational position, with a clear preference for individuals in leadership roles. Interview scheduling was centrally coordinated across all organizational contexts, and communication with participants occurred primarily through hierarchical channels rather than directly with participants. This is illustrated by access to the military context. Two days after the gatekeeper proposed embedding interviews within an internal training session, I received the following message: “The schedule has been finalized, including the assignment of participants to the interviews. … Details regarding the military personnel can be found on the final page of the attached schedule. I hope this works for you. …” (gatekeeper email, military institution). The attached document listed dates, time slots, and surnames. The resulting interview sample consisted exclusively of male, high-ranking soldiers in leadership positions.

#### 4.1.2 Hierarchical reproduction of access and information control

This selective approach was reproduced across hierarchical levels through a cascading form of access control. Initial agreements with high-level gatekeepers did not translate into neutral access at lower organizational levels. Instead, access was repeatedly re-mediated as information and authority were passed down through the hierarchy. Employees in leadership roles at different levels were involved in identifying, informing, and pre-selecting potential participants within their teams.

As we parted ways, I asked whether he [high-ranking employee and first contact person] might invite colleagues to participate and offered to send an email with the invitation and contact details. Before I could write it, he had already recruited two colleagues and suggested date, time, and location. … (field notes/postscript, police)

This mode of selection suggests that participation was not solely based on individual willingness but embedded in organizational logics of representation and control.

#### 4.1.3 Pre-structuring research interactions

Control over access also extended into the informational framing of the research encounter itself. In several instances, participants received prior briefings that selectively re-framed the study beyond the information originally provided for recruitment purposes. These briefings emphasized certain aspects of the research and introduced anticipatory framings of its purpose, shaping how the topic was understood before the actual encounter. This is illustrated by an interview conducted at a police station. Upon arrival, I was welcomed by a First Chief Police Commissioner who presented a series of pre-formed interpretations and normative assessments of contemporary policing practices:

I had little opportunity to introduce myself, as he immediately referred to the research exposé handed to him by his superior—without my knowledge and not intended for participant recruitment. He made two points right away: first, that a policeman who simply follows orders is outdated, especially with cell phone cameras everywhere; second, that wearing a uniform is largely meaningless due to variations in components. As he spoke, I reflected on how to handle … his assumptions about my intentions. … (field notes/postscript, police)

This moment of in-situ reflection illustrates how researcher positioning is not external to ODR dynamics but embedded within them: the uncertainty about how to respond to the commissioner’s pre-framing was itself a product of the power asymmetry structuring the encounter, and attending to that uncertainty became an analytical resource.

The consequences of the pre-structured research interactions became visible in recurring questions and statements before, during, and after interviews, for example: “Will my supervisor see what I said?,” “How will this be reported back to the department?,” or “I’m here because my boss asked me to participate” (composite fieldnote excerpts across all three organizational contexts). A central feature of this dynamic was the absence of direct contact prior to the interviews, preventing independent rapport-building outside organizational hierarchy.

### 4.2 Control over the research topic’s perspectivation

Control over the research topic’s perspectivation emerged as a second central mechanism through which organizations shaped the research process. Variation in how participants framed the topic across organizational contexts revealed a consistent pattern of structured organizational control rather than idiosyncratic differences in interpretation.

#### 4.2.1 Depoliticization of the research topic

Composite narratives—a methodological approach to present interview findings by synthesizing individual accounts into collective patterns (Willis, 2019)—across police, military, and clinical settings reveal two dominant strategies of depoliticization. First, temporal distancing was used to frame destructive obedience as a relic of a bygone, authoritarian era. Participants frequently contrasted “rigid structures” of the past with today’s “collegial tone” and “mission-type tactics,” arguing that institutional safeguards—like flatter hierarchies and the formal obligation to remonstrate—now make blind obedience structurally impossible. In this framing, obedience-related abuses were positioned as incompatible with present-day organizational norms (e.g., “This rigid structure has been relaxed…,” “Traits like that do not belong here anymore”). Second, discursive reframing was employed to neutralize hierarchical dynamics. The concept of “order” was mentioned to be “hardly present” and replaced by “bottom-up consultation,” where decisions were described as being “delegated upwards” for support. Even when formal hierarchies remained visible—for instance, when supervisors held the “final say”—these moments were not framed as obedience but as necessary “professional coordination.”

These patterns suggest that destructive obedience is rendered epistemically marginal, thereby increasing the likelihood that critical findings are attributed to a misalignment between the research focus and organizational context.

#### 4.2.2 Narrative control

The interviews revealed three recurring patterns of narrative control: contradictions within individual narratives, discrepancies between on- and off-record accounts, and systematic variation depending on modes of access.

Contradictions emerged within individual interviews. Participants frequently downplayed the relevance of hierarchical authority, obedience, or the symbolic power of organizational markers such as uniforms. At the same time, their accounts contained elements that implicitly reaffirmed these dynamics. For instance, one senior police officer repeatedly described the uniform as “meaningless,” while simultaneously acknowledging that it shaped his behavior in public:

When I’m walking down Street B as a civilian … I’ve got a cigarette hanging out of the corner of my mouth … And if I’m walking down there in uniform, I personally would never smoke a cigarette. If I ever had to …, then (laughing) it would go straight into the nearest trash can, of course.

As shown by composite narratives across police, military, and hospital settings, claims that obedience or misuse of power were irrelevant coexisted with concrete descriptions of hierarchical decision-making, sanctioning practices, and career-related consequences in cases of questioning an authoritarian order. For example, one superior reported that a subordinate who openly challenged an order was reassigned to “office work for a quarter of a year for that,” while others noted that employees often remained silent “because they did not want any trouble.”

A second pattern took the form of a dialectic between on- and off-record accounts, documented in fieldnotes capturing off-record interactions. While on-record narratives were often characterized by silence, denial, or the absence of morally problematic situations, off-record conversations frequently produced more detailed and ambivalent descriptions. Participants described, for instance, participation in informal sanctioning practices, morally contested decisions in military operations, ignoring the results of risk assessment, patient over- or undertreatment. In several cases, such issues were briefly alluded to during recorded interviews but only elaborated upon once the recording had ended.

Discrepancies between discourse and practice also became visible in field observations, despite their prior discursive downplaying. For example, during a clinical job shadowing, a suggestion to anonymize patient data (which should have been mandatory under data protection regulations given my presence) was dismissed by a senior physician (field notes, first job shadowing, university hospital). Similarly, organizational practices were at times actively staged in ways that shaped how I could observe and interpret the field. In the police context, for example, I was invited to accompany officers in a patrol vehicle on the grounds that this was necessary to “understand the realities of the job” before conducting interviews. While not part of the original research design, this arrangement functioned as a precondition for meaningful engagement and effectively curated a particular representation of organizational practice for observational purposes. It also involved a degree of informal flexibility that would not normally have been granted under standard procedural rules (field notes, second job shadowing, police). Recognizing this arrangement as a staged representation rather than a neutral field encounter required reflexive attention to the organizational interests shaping the conditions of access—an interpretive stance that itself emerged through reflexive awareness of my positioning as a managed audience.

The third pattern of narrative control is reflected in the tone and content of interview narratives. Participants recruited through formal hierarchical channels—particularly subordinates—exhibited patterns of compliance-oriented communication. As shown by a narrative composite across police, military, and clinical settings, their narratives were often brief and defensive, characterized by evasive linguistic markers such as: “I have not given it any thought yet, so I’m unable to comment.” Field observations suggest that for this group, the researcher was inseparable from the organizational frame; their primary concern was the flow of information back to superiors (“Will my supervisor see what I said?,” “The department head approached me with the idea”). Off-record conversations were rare. In contrast, senior personnel nearing retirement felt relationally secure enough to engage in the dialectic of on-record and off-record narratives described earlier. Participants accessed through informal channels provided comparatively more detailed and critical accounts. These included, for example, descriptions of having been unjustifiably sanctioned, being pressured to monitor or marginalize inconvenient colleagues, or being pressured to cause harm and/or disadvantage to addressees (e.g., by patient overtreatment or racial profiling), often as a means to achieve organizational goals, meet performance targets, or comply with supervisors’ orders.

These differences suggest that access modes are associated with narrative control and openness. This interpretation did not emerge from single-case interpretation but from sustained comparison across police, military, and university hospital contexts, where similar access-dependent patterns recurred despite differences in institutional settings. The cross-case convergence made it increasingly difficult to maintain an individual-level explanation of participants’ narratives, shifting the analytic focus toward structurally conditioned disclosure regimes.

### 4.3 Control over research design

Control over research design emerged as a third mechanism through which organizations shaped the research process. This was particularly evident in relation to the planned role-play, which was initially approved but subsequently modified, constrained, or rendered unfeasible across organizational settings. The repeated need to renegotiate core elements of the role-play made it increasingly difficult to maintain a distinction between conducting the research and negotiating its conditions. Attending to this blurring revealed the extent to which methodological decisions had themselves become subject to organizational influence.

#### 4.3.1 Withdrawal and gate closure

In the police context, initial high-level approval and openness toward implementing the role-play shifted into withdrawal once methodological details were specified. After concrete proposals regarding participant numbers, procedural design, and organizational integration had been discussed directly with the responsible police commissioner, subsequent communication was increasingly delayed and eventually discontinued. Subsequent contacts involved stalling tactics, such as citing office relocations: “Due to an office relocation, I was unavailable via email for an extended period of time. …” (gatekeeper email, police). Despite final assurances that dates for the study would be provided, the organization ceased all communication, leaving follow-up messages unanswered. This amounted to an implicit veto through inaction.

#### 4.3.2 Methodological reconfiguration

Within the setting of the university hospital, methodological control emerged as a reconfiguration of the role-play. Despite having secured an initial agreement for a pretest role-play from a gatekeeper in a leadership role, the actual conduct diverged from the agreed-upon procedure. In the final email exchange prior to the pretest session, I received the following information: “Honestly, it would be more practical and nicer if we could stay together as a group and then switch roles. But if you’d prefer to do it differently, that’s fine too.” (gatekeeper email). On the day of the pretest session, I presented the procedure and pre-constructed role-play scenarios. Subsequently, the gatekeeper notified me, on behalf of the team, of their decision to discontinue the previously proposed scenarios. Instead, an on-site negotiation unfolded, during which the group discussed possible alternatives and ultimately agreed to develop their own role-play scenario. This scenario was based on a real team conflict that had previously occurred and was collectively adapted for the purposes of the exercise (field notes, pretest role-play). This reconfiguration was justified as increasing realism and improving team fit. Consequently, the original research design was displaced, and the participating organizations assumed control over methodological decisions. Subsequent attempts to extend the role-play stalled.

#### 4.3.3 Methodological redefinition

In the military context, the initial pretest role-play was conducted within an internal training environment managed by organizational instructors and largely followed the planned procedure (field notes pretest role-play).

Efforts to plan the subsequent data collection were met with strategic scientization. Gatekeepers demanded a shift toward a classical experimental logic, while critiquing the original role-play as “overly complex” and “unlikely to produce valid effects.” This critique served as a lever to push for a standardized vignette-based design to address logistical and participant-related constraints: „In summary, we therefore propose simplifying the experiment,” or “I am glad and relieved that our idea of using a photo story or video did not catch you off guard. You are correct in understanding that this would involve a standardized vignette-based survey” (gatekeeper emails). By making continuation contingent on this adjustment, the organization effectively redefined the permissible conditions of data production—even though the proposed survey was ultimately not implemented.

### 4.4 The cascading role of relational dynamics in organization-driven research

Organization-driven research is characterized by the interdependence of organizational control mechanisms. The ability to control access determined who could be approached, while control over information shaped the research framing before interaction. These conditions determined the research topic’s perspectivation and extended into relational dynamics, which served as a transmission mechanism for organizational control during interaction. Participants recruited through formal channels provided more cautious accounts, whereas those accessed informally articulated more critical experiences. These differences in participants’ narrative openness reflect their relational positioning within organizational power structures.

In cases where participants were recruited on an informal basis, relational dynamics frequently extended beyond differences in disclosure and actively reconfigured the research interaction. In several cases, interviewees articulated grievances or unresolved conflicts, positioning the researcher as a means of ganing visibility. This phenomenon is exemplified by a case in which a participant in a legal dispute reframed the interview to document and publicize their experience of workplace conflict: “First and foremost, I’m reaching out not because I’m worried about the success of your research, but because I want to share the story of the mobbing I experienced. It’s not over yet …” (follow-up email, participant in legal dispute).

These patterns show that relational power dynamics actively structure what is articulable in research settings. Leadership shapes subordinate behavior, formal recruitment embeds accountability, and informal access relaxes constraints. Thus, relational dynamics translate organizational control over access into narrative performance. These interventions affect methodological design, requiring ongoing adjustment. By regulating sample composition, heterogeneity, and communication channels, organizational control influences data generation, shaping the research process and its outcomes. Consequently, aspects of researcher autonomy became subject to organizational influence.

Overall, organizational influence operated as a cascading process continuously structuring interaction and knowledge production, thereby recursively reproducing organizational power structures.

## 5 Organization-driven-research as organizational impression management

The multifaceted manifestations of ODR suggest a systemic organizational effort to regulate how the organization is perceived during the research encounter to maintain a favorable image and limit exposure to discrediting interpretations. This paper interprets these dynamics accordingly as a manifestation of o*rganizational impression management (OIM)*. Organizational impression management is defined as purposeful organizational actions or spokesperson performances to influence how audiences perceive an organization as an entity (Mohamed et al., 1999). While assertive OIM strategies are oriented toward proactively constructing a favorable organizational image, defensive strategies aim at denying, distancing from, or repairing a potentially damaging situation or event (Bolino et al., 2008; Mohamed et al., 1999). Such strategies become particularly salient under conditions of potential reputational threat (Stratulat, 2019).

Within research on sensitive issues, OIM is enacted through organizational influence on research interactions, including the framing of participant accounts and the structuring of encounters. The researcher thereby becomes a central, and often critical, audience, which transforms organizational participation in research into a strategic process of impression management.

The interpretation of ODR as OIM is itself a theoretical choice shaped by the researcher’s own positionality: entering hierarchical organizations as an external researcher with a visible research agenda made the impression management dynamics analytically salient in ways that might be less visible to insider researchers embedded in the same relational structures.

**Figure 1:**
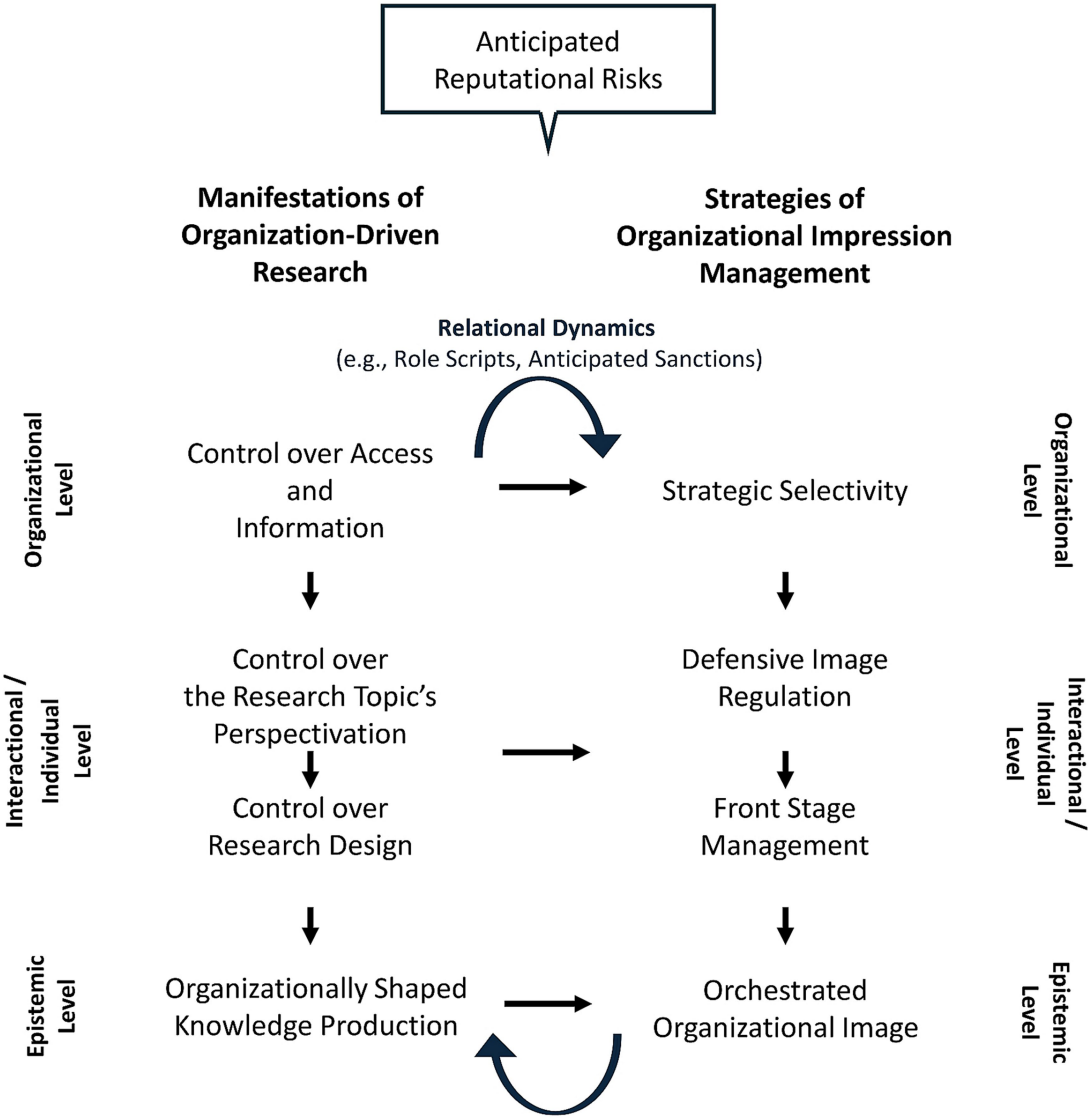
Conceptual model of organization-driven research (ODR) as organizational impression management (OIM).

The following sections develop this interpretation by discussing how organizational impression management unfolds across organizational and individual levels. Figure 1 illustrates the underlying theorizing visually.

### 5.1 Organization-driven research as a manifestation of organizational impression management at the organizational level

A primary dimension of OIM in ODR contexts is the strategic regulation of access and information. As shown in the manifestations of strategic gatekeeping and participant pre-selection, access was systematically mediated through hierarchical structures, including the pre-selection of interviewees, centralized coordination, and restricted direct contact between researcher and participants. From an impression management perspective, this reflects strategic selectivity, through which organizations regulate who becomes visible as an authorized spokesperson (Aké and Boiral, 2023). Building on Elsbach and Sutton (1992), these spokespersons can be understood as carriers of organizational accounts, that is, strategically constructed representations that align the organization with preferred identity claims. This structural control extends into the pre-structuring of research interactions. As illustrated above, participants often entered the interview situation with prior briefings or mediated framings of the research topic. These anticipatory interpretations function as pre-interactional information control (Bolino et al., 2008), delimiting the boundaries of what participants perceive as safe to disclose.

Control over the research topic’s perspectivation constitutes a second dimension of OIM, primarily enacted through defensive image regulation. As shown in the manifestations of depoliticization, destructive workplace obedience was recurrently framed as outdated, exceptional, or no longer relevant to contemporary practice. Participants contrasted “rigid structures” of the past with present-day “collegial” or “mission-type” practices and referred to institutional safeguards to support this interpretation. From an OIM perspective, these narratives can be interpreted as normalizing accounts (Elsbach, 1994), which render potentially problematic practices less threatening. A related form of discursive impression management emerges in the redefinition and neutralization of hierarchical relations. As documented in the manifestations, formal “orders” were frequently described as largely absent and replaced by notions such as “bottom-up consultation,” while authoritarian decision-making was reframed as “professional coordination.” Even where hierarchical asymmetries remained visible—for instance in references to the “final say” of senior professionals—these were not articulated in terms of obedience. This practice serves image-enhancing and blame-avoiding functions under conditions of potential reputational threat (Bolino et al., 2016; Martins et al., 2021).

Finally, OIM manifests in control over methodological design. As shown in the manifestations of withdrawal, reconfiguration, and redefinition, the originally planned role-play was repeatedly modified, constrained, or rendered unfeasible once the research design moved from abstract approval to concrete implementation. Compared to more structured formats such as guided interviews, interactive methods like role-play introduce greater variability in participant responses and reduce the predictability of the results obtained (Stokoe, 2013). From an impression management perspective, the observed adjustments can therefore be interpreted as defensive impression management strategies aimed at limiting less controllable forms of interaction (Bolino et al., 2008). This aligns with Goffman’s (1959) notion of front stage management, in which actors curate those aspects of interaction that are visible and consequential for external evaluation (Pernelet and Brennan, 2023).

In several cases, organizational practices were not only described but actively staged, for instance by embedding the researcher in curated observational settings such as ride-alongs or by requiring the researcher to engage in organizational routines as a precondition for conducting interviews. Thus, the researcher did not remain external to these dynamics but became incorporated into the logic of impression management as a situated audience (Duarte, 2011; Klitgaard et al., 2021). As a result, organizations retain control over the range and type of narratives available for analysis and align potentially sensitive aspects with a legitimate and favorable self-presentation (Bolino et al., 2016; Perkiss et al., 2021).

In line with prior research, these OIM practices appear to be driven by an underlying anticipation of reputational risk and a loss of control over how the organization may be represented (Benoit, 1997; Gřundělová et al., 2025; Hood, 2011).

### 5.2 Organization-driven research as a manifestation of organizational impression management at the individual level

Building on Bourgoin and Harvey (2018), organizational control over access, perspectivation, and research design translates into individual-level practices of OIM through role-specific scripts. Organization-driven research is therefore to be understood as an interactional setting in which organizational members—as emphasized by Lamertz (2022)—perform and reproduce organizational identity claims “to orchestrate a coherent impression” (p. 3) regardless of whether these claims fully correspond to their personal beliefs. This becomes visible in practices of depoliticization and narrative control through which organizational members align their self-presentation with broader organizational narratives. Particularly the outlined dialectic of on- and off-record narratives indicates that organizational members “authenticate formal organizational claims by projecting roles … that corroborate authenticity” (Lamertz, 2022, p. 31). For example, one participant in a leadership position simultaneously emphasized “flatter hierarchies” while acknowledging the existential dependence on performance evaluations:

First of all, we have flatter hierarchies than in the past, but in most cases, we still want to be promoted. And that depends on my performance evaluation … And if you do not want to, you do not have to. But (laughs) then one can copy his pay slips until he retires.

Combining elements of hierarchical softening with references to continued career dependence enabled the maintenance of surface-coherence with organizational accounts (Bolino et al., 2016). Organizational members therefore actively structure what is emphasized, downplayed or rendered irrelevant to elicit positive audience responses (Bolino et al., 2008). This micro-level agenda-setting (Jacobsson and Åkerström, 2013)—including the staging of routines like ride-alongs—positions the researcher as a situated audience whose perception must be managed (Duarte, 2011; Klitgaard et al., 2021).

The orchestration of a coherent impression at the intersection of organizational and individual level is contingent upon perceived authenticity (Lamertz, 2022). Organizational credibility depends on the perceived alignment between formal claims and members’ performances. The reported contradictions between on- and off-record communication, as well as variations in narrative depth across participant groups, indicate that such coherence is not pre-given but situationally produced in interaction. This interpretation aligns with Ashforth et al. (2020), who suggest that discrepancies between experienced and projected organizational realities may remain publicly unarticulated while still affecting underlying identification processes.

With Gřundělová et al. (2025), employees’ contribution to the orchestration of a coherent organizational impression can be interpreted as an adaptive response to the anticipation of sanctions. Participants’ references to evaluation systems, career dependencies, and consequences for challenging authority indicate that communicative behavior is embedded in perceived risk. According to Bolino et al. (2016), organizational members become sensitive to how disclosures may be evaluated by superiors, prioritizing image-safe information over potentially discrediting accounts. Gatekeeping structures, hierarchical recruitment, and pre-structured interactions thus signal implicit expectations regarding appropriate conduct.

Relational dynamics function as a key transmission mechanism linking OIM at the organizational and the individual level. Variations in narrative openness across hierarchical positions and recruitment channels reflect differing exposure to accountability and sanction risk. Participants recruited through formal channels tend to provide cautious and minimally elaborated accounts, whereas informally accessed or more secure participants display greater openness. These patterns can be understood as contextually structured enactments of defensive impression management, in which withholding, downplaying, or selective disclosure protects both individual standing and organizational image (Bolino et al., 2008; Bolino et al., 2016).

Relational dynamics also shape how participants orient themselves toward the researcher. Participants may position the researcher as a specific type of audience—for example, as a trustworthy confidant, a potentially risky external observer, or a conduit for external visibility—and adjust their communicative behavior accordingly (Gřundělová et al., 2025; Othman and Abdul Hamid, 2018). Such practices do not stand outside ODR but reflect its underlying power structure: where internal voice is constrained, the research encounter may function as an alternative channel for expression that partially circumvents hierarchical control. This becomes evident, for instance, in systematic differences across recruitment modes: participants recruited through formal channels tended to shift sensitive content to off-record interactions, whereas informally recruited participants were more likely to articulate grievances and critical experiences on record. The contrast between alignment and deviation thus reflects varying exposure to organizational authority and illustrates how interactional behavior is conditioned by the same relational dynamics that sustain organizational control.

Overall, ODR unfolds as a recursive multi-level process in which structural interventions shape interactional behavior, while individual performances simultaneously reproduce organizational-level OIM.

## 6 Discussion

Building on a phenomenon-based theorizing approach, this study examines how investigating sensitive issues in organizational contexts—illustrated here through destructive workplace obedience—reveals complex and systematic patterns of organizational influence on the research process. Prior literature has addressed the dynamics documented here—such as control over access and information, control over the perspectivation of the research topic, or control over research design—as isolated methodological challenges to be managed (Americo et al., 2024; Crowhurst and Kennedy-Macfoy, 2013; Kay, 2019; Monahan and Fisher, 2015). However, the present paper argues that these dynamics constitute a patterned and multi-level phenomenon—termed *organization-driven research*—in which multiple forms of organizational influence emerge simultaneously, interact with one another, and are shaped by participants’ embeddedness in organizational hierarchy. Prior discourse on gatekeeper control implies that organizational influence can be navigated through credibility management, relationship-building, or strategic communication (Americo et al., 2024; Brannick and Coghlan, 2007; Kay, 2019; Reeves, 2010). The concept of ODR extends this perspective by demonstrating that organizational influence does not cease once formal access has been granted and maintained throughout data collection. Rather, it operates throughout the research process—shaping participant selection, framing research encounters, and constraining methodological design. Organizational influence thus operates as a structuring force that shapes how organizational realities are accessed, interpreted, and represented in research on sensitive organizational issues.

Theorizing ODR as a manifestation of organizational impression management provides a theoretical account of why organizations and their members engage in these practices: organizational control mechanisms function as coordinated efforts to mitigate reputational risk and stabilize a favorable organizational image (Bolino et al., 2016; Scheytt and Pflüger, 2024). Thus, ODR represents a form of epistemic impression management through which organizations seek to retain control over the conditions under which organizational realities become knowable through research on sensitive issues. Prior OIM research has predominantly examined how organizations manage their image through external communications, crisis responses, annual reports, and spokesperson performances directed at audiences such as investors, regulators, or the general public (Bolino et al., 2016; Elsbach, 1994; Mohamed et al., 1999). The present study extends this body of work by demonstrating that researchers constitute a specific and consequential audience for OIM. While multi-level dimensions of OIM have been theorized in prior work—for instance in terms of how individual spokespersons enact organizational identity claims (Lamertz, 2022) or how role-based performances sustain organizational narratives (Carroll and Wheaton, 2009)—empirical documentation of how organizational impression management strategies cascade across organizational, interactional, and epistemic levels within a single research project remains limited. This paper addresses this gap by demonstrating how such cascading dynamics unfold in research on sensitive organizational issues. Organizational-level practices—such as strategic gatekeeping, participant pre-selection, and the pre-structuring of information—extend into the research encounter and are reproduced at the level of situated interaction. At the individual level, these structural conditions translate into role-based performances, in which participants orient their accounts toward perceived expectations of organizationally appropriate conduct—ultimately shaping the conditions under which organizational knowledge on sensitive issues is produced during a research encounter.

This translation is crucially mediated by relational dynamics, where differences in narrative openness, shifts between on- and off-record communication, and variations across recruitment modes indicate that participants’ communicative behavior is deeply shaped by their position within relations of authority, accountability, and dependence. These findings are consistent with the concept of organizationality (Scheytt and Pflüger, 2024), which posits that research in organizations is fundamentally structured by specific institutional features: hierarchy, formal membership, and institutionalized control mechanisms. While the qualitative methods literature has long recognized that participants engage in individual-level impression management (Jacobsson and Åkerström, 2013), ODR demonstrates that such performances are not reducible to personal motivation—they are structurally conditioned by hierarchical accountability and organizational membership. The formal accountability and hierarchical oversight inherent in organizational life determine the interactional conditions under which participants feel authorized or safe to speak. Consequently, the anticipation of professional sanctions or reputational risks fosters an alignment with organizational narratives, increasing the likelihood that individual narratives remain within organizationally permissible scripts—dynamics that individual-level accounts of impression management cannot fully capture.

Yet the dynamics of ODR also point to an underlying tension: the role of power and authority in shaping how organizational realities become knowable in the first place. Organizational influence in research settings does not operate primarily through explicit coercion, but through the structuring of conditions under which participation, disclosure, and articulation become possible (Clegg, 2009; Lukes, 2005). In contexts characterized by hierarchy and accountability, what can be said is less a matter of individual willingness than of relational positioning and anticipated consequences. This points to a *control–exposure paradox*: the very mechanisms through which organizations seek to control their external representation may simultaneously render their internal dynamics visible. Organizational impression management may inadvertently expose the very structures of authority and compliance it seeks to regulate. In this sense, organizational impression management does not merely limit knowledge production; it actively shapes the conditions under which its own limits become analytically traceable.

Taken together, these insights advance current knowledge in several respects. *Theoretically*, ODR introduces a conceptual vocabulary for phenomena that have hitherto been described but not explained. *Epistemologically*, theorizing ODR as manifestation of OIM reveals that the conditions of knowledge production about organizations are themselves organizationally shaped, with consequences that extend beyond the research encounter itself. One such consequence concerns the conditions under which research-generated knowledge is interpreted, disseminated, and incorporated into organizational practice. Research on organizational knowledge translation suggests that whether findings become integrated into collective organizational intelligence primarily depends on the social processes through which they are interpreted and legitimized (Ahn and Hong, 2019; Oborn et al., 2013; Salter and Kothari, 2016). Accordingly, ODR may reproduce established organizational narratives rather than enable the generation of the critical knowledge necessary for adaptive learning and organizational self-reflection, identifying unknown unknowns, and open innovation (Crossan et al., 1999; Chesbrough and Bogers, 2014). Fields such as audit, governance, and compliance have developed procedural safeguards—including independence requirements, structured verification procedures, and conflict-of-interest protocols—to preserve evaluative credibility and legitimacy (García-Hernández et al., 2023; Power, 2013). These practices may offer a valuable reference point for future theorizing on how governance structures and knowledge-sharing arrangements can enable openness to critical scrutiny despite reputational concerns. At the same time, the increasing use of AI-supported monitoring and evaluation systems may substantially transform the conditions under which ODR dynamics unfold: where surveillance is intensified and sanction risks are reshaped, the threshold at which organizational members consider experiences safe to articulate may shift accordingly (Anjelia et al., 2025; Chugunova and Sele, 2022; Xavier and Korunka, 2025). *Methodologically*, reframing organizational influence as a constitutive rather than contingent condition of research practice shifts the analytical task from managing challenges toward engaging with relational dynamics, narrative inconsistencies, and access patterns as data on the organizational logic under investigation.

The following section develops the implications of this reframing for research practice.

### 6.1 Methodological implications of organization-driven research

First, the findings indicate that organizations might perceive research on sensitive issues as direct inquiries *about* their specific conduct. Addressing organizations not as objects of evaluation but as contextual environments that provide structural conditions for research participation can help reduce such defensiveness and facilitate access (McGregor, 2023). By decoupling the unit of recruitment (organizations) from the unit of analysis (individual participants), researchers can frame the organization not as the primary object of evaluation, but as a methodological gateway that provides the necessary structural conditions for research (e.g., hierarchy and membership).

Second, variation in access emerges as methodologically consequential. Differences between formal and informal recruitment are associated with systematic variation in response patterns, suggesting that access is not merely a logistical precondition but a constitutive element of data production (Riese, 2019). Employing multiple access modes enables researchers to capture a broader spectrum of organizational representations and to identify systematic differences in how organizational realities are articulated under varying degrees of formal control (Klitgaard et al., 2020; Lamertz, 2022).

Third, relational dynamics—such as strategic gatekeeping, selective disclosure, or narrative control—should be interpreted as analytically meaningful epistemic signals rather than mere methodological challenges. Such patterns may mirror a broader climate of organizational silence, where the withholding of information is a collective protective strategy driven by a lack of psychological safety (Dupret, 2019; Morrison and Milliken, 2000). Attending to these patterns makes the unsayable analytically visible through the very act of its suppression.

Fourth, ODR as a manifestation of organizational impression management reframes research encounters as sites of role performance. Participants often act as representatives of their organizations, scripting narratives according to organizational expectations of appropriate conduct—expectations that constrain not only what can be said, but what can be deemed as sayable (Carroll and Wheaton, 2009; Lamertz, 2022; Scheytt and Pflüger, 2024). Inconsistencies, shifts in narrative framing, or contradictions within narratives should therefore be treated as data on how OIM is accomplished in interaction. Building on this perspective, methodological practice shifts toward a more reflexive and analytically engaged stance toward impression management dynamics in the field. An ethnographic orientation can further extend this approach by situating interviews within broader patterns of observed organizational practice (Duarte, 2011; Klitgaard et al., 2021). Combining interview data with observations allows tensions between front-stage narratives and enacted practices to become analytically visible (Kostera and Harding, 2021). Rather than adopting a passive role, researchers can assume a critical audience position that treats the interview as a site of negotiation in which organizationally structured scripts are both enacted and potentially subject to situational reconfiguration. The literature on qualitative interviewing offers established techniques for probing beyond rehearsed accounts, including structured forms of probing (Robinson, 2023; Gerson and Damaske, 2020), digging deeper (Holliday, 2012), and the analytical use of inconsistencies (Watson, 2006), which reframe contradictions as indicators of shifting positionalities within organizationally structured accounts. Thus, the interview itself becomes primary data for analyzing the “methodological impression management” inherent in the field (Gengler and Ezzell, 2018). This also requires that researchers attend reflexively to their own positioning within the research encounter, recognizing that their interpretations of narrative inconsistencies or relational dynamics are themselves shaped by the conditions of access and the interactions that preceded them (Olmos-Vega et al., 2023).

Fifth, the previously outlined dynamics are further shaped by the spatial setting of data collection. Data collection within organizational settings may reinforce role-based performances and hierarchical accountability relations, whereas external or neutral locations can partially loosen these constraints and may facilitate the expression of personal experiences, attitudes, and values. Bilsland and Siebert (2024) demonstrate that shifting the interview setting can reverse power relations, with places functioning as prompts that yield insights into otherwise inaccessible organizational phenomena.

Taken together, these methodological implications suggest that ODR is a constitutive condition of knowledge production in organizational research. Rather than assuming that methodological challenges can be fully mitigated, the findings indicate that they are inherent to the research process itself.

### 6.2 Directions for future research and limitations

If organizations systematically shape the conditions under which they become knowable to external researchers, repeated dynamics of ODR may reproduce curated narratives across studies, reports, and evaluations—reinforcing particular understandings while marginalizing contradictory accounts. Research on organizational impression management has shown that organizational self-presentations may become progressively detached from the conditions they claim to describe (Boiral, 2013; Talbot and Boiral, 2015). This possibility resonates with debates on simulacra and hyperreality, according to which representations may become self-referential and accepted as reality even when disconnected from what they claim to represent (Baudrillard, 1994; Boje, 2002; Grandy and Mills, 2004). Future research should therefore examine whether accumulated forms of ODR contribute to the emergence of simulacra-like representations of organizational reality.

These considerations have important ethical implications. Prior discourse has emphasized that qualitative researchers must balance the pursuit of academic rigor with the obligation to protect participants who may fear risks related to anonymity, confidentiality, and potential professional or personal sanctions (Bell and Bryman, 2007). In parallel, scholars have highlighted that research on sensitive organizational issues may also entail risks for organizations themselves, including potential reputational damage and threats to institutional integrity (Canadian Institutes of Health Research, Natural Sciences and Engineering Research, and Social Sciences and Humanities Research Council of Canada, 2022; McGregor, 2023). Building on Scheytt and Pflüger’s (2024) concept of organizationality, the dynamics of ODR reveal that ethical tensions—such as layered field access, power asymmetries, and sanction risks—emerge throughout the research process. These tensions influence voluntariness, confidentiality, and protection from harm and are embedded in, and potentially constrained by, the characteristics of the respective organization. This calls for a more process-oriented and context-sensitive understanding of research ethics in organizational settings (Fecke et al., 2022; McGregor, 2023), in which ethical conditions are treated as empirically constituted within organizational contexts rather than normatively assumed. This thereby opens a promising avenue for future research into the ethical dynamics of organization-driven research.

Despite its contributions, the conceptualization of ODR as a manifestation of organizational impression management is subject to several limitations.

First, organizational impression management integrates multiple theoretical traditions, including interactionist, institutional, and legitimacy perspectives (Bolino et al., 2016; Suchman, 1995), which implies that its underlying mechanisms are not fully unified. While this hybridity enables analytical flexibility, it also means that ODR represents a synthesized interpretation rather than a consolidated theory of organizational influence. Moreover, related perspectives from decision theory or moral psychology may offer complementary explanations of ODR (Caspar, 2024; Leary and Kowalski, 1990). Hoque et al. (2013) demonstrate that using theories with epistemological tensions, captured by appropriate research methods, enables researchers to explore different and sometimes contradictory layers of organizational realities.

Second, there is a risk of over-attributing strategic intent to all forms of organizational resistance, filtering, or inconsistency. Distinguishing between routine organizational contingencies and systematic impression management remains methodologically challenging (Bolino et al., 2008; Ferris et al., 2018). At the same time, individual participation in OIM cannot be reduced to structural compliance alone. Participants may address multiple audiences simultaneously, aligning with organizational expectations while also safeguarding their own professional identity to avoid negative social evaluation (Harrison et al., 2018; Leary and Kowalski, 1990).

Third, the conceptualization of ODR is based on observable manifestations of organizational control. In cases where such control is highly effective—such as through non-participation or extensive organizational silence—ODR may leave only limited empirical traces (Morrison and Milliken, 2003). As a result, the present conceptualization of ODR captures visible expressions of organizational influence rather than its full extent.

Fourth, ODR is derived from a single research trajectory situated in a specific empirical context characterized by hierarchical organizations. Its applicability to less formalized or more fluid organizational settings therefore remains an open question. In such contexts, impression management dynamics may operate through more decentralized or peer-based mechanisms, potentially altering the cascading patterns observed in this study (Barsness et al., 2005). Beyond less formalized organizational settings, future research should examine whether similar dynamics emerge in other forms of organizational knowledge production—including internal surveys, consulting assignments, audit procedures, and governance reviews—where knowledge producers may themselves be organizational members subject to analogous accountability pressures.

Fifth, the study focuses on destructive workplace obedience as a specific type of sensitive research topic. Prior work suggests that methodological challenges and effective research strategies can vary depending on the nature of the topic under investigation (Jehn and Jonsen, 2010; Saharan et al., 2021). The control mechanisms identified in this study may therefore not translate directly to other sensitive issues, where distinct forms of risk perception and OIM responses may prevail. As Piening et al. (2020) demonstrate, organizational identity threats produce variable employee responses depending on the nature of the threat, suggesting that the dynamics of organizational defensiveness are topic-contingent rather than universal.

Sixth, a primary limitation is that ODR emerged as a by-product of a study originally designed to investigate workplace obedience. While methodological insights frequently emerge during the course of research projects (Langley, 2021), such serendipitous discoveries remain shaped by the original design that produced them (Fine and Deegan, 1996), suggesting that the research design was not originally optimized to capture ODR dynamics.

Taken together, these limitations point to a productive research agenda: future work should examine organization-driven research as an empirical phenomenon in its own right, extending the analysis beyond the contexts and methodological conditions of the present study to investigate how organizations shape not only what is known about them, but the conditions under which knowledge production on sensitive organizational issues becomes possible.
